# Can the two-point method based on peak and trough concentrations accurately estimate the area under the curve of polymyxin B? A Monte Carlo simulation study

**DOI:** 10.3389/fphar.2026.1838789

**Published:** 2026-06-24

**Authors:** Jieqiong Liu, Jianping Zhu, Gang Liang, Yi Yang, Lingyan Yu, Yuhua Zhao, Zhenwei Yu

**Affiliations:** 1 Department of Pharmacy, Sir Run Run Shaw Hospital, School of Medicine, Zhejiang University, Hangzhou, China; 2 Research Center for Clinical Pharmacy, College of Pharmaceutical Science, Zhejiang University, Hangzhou, China; 3 Department of Pharmacy, The Second Affiliated Hospital, School of Medicine, Zhejiang University, Hangzhou, China; 4 Affiliated Xiaoshan Hospital, Hangzhou Normal University, Hangzhou, China

**Keywords:** area under the curve, polymyxin B, population pharmacokinetics, trapezoidal method, two-point method

## Abstract

**Background:**

The area under the concentration‒time curve (AUC) is recognized as the therapeutic drug monitoring index of polymyxin B. The two-point method, which is based on peak and trough concentrations, is recommended for AUC estimation. However, its accuracy needs to be fully elucidated.

**Methods:**

A Monte Carlo simulation study was performed on published population pharmacokinetic (PPK) models. A series of plasma concentrations at steady state were simulated for each scenario. The performance of the two-point method was evaluated via mean absolute percent error (MAPE) and root mean square error (RMSE), with acceptable performance limits defined as ≤15% for the MAPE and ≤20% for the RMSE. Additionally, variations in infusion duration, body weight, and creatinine clearance were also investigated.

**Results:**

In the one-compartment model, the two-point method exhibits excellent predictive performance, characterized by both the MAPE and RMSE < 15%. For multicompartment models, prolonging the infusion duration markedly improved the accuracy of the two-point method, as demonstrated by a decrease in the average MAPE from 30.2% to 7.78% and in the average RMSE from 35.5% to 9.12% when comparing infusion durations of 0.5 h and 2 h. Body weight and creatinine clearance had limited influences on estimation accuracy in most models.

**Conclusion:**

The two-point method, which is based on peak and trough concentrations, represents a strong pharmacokinetic rationale and generate a testable hypothesis for the clinical application for estimating the AUC of polymyxin B. We suggest prolonging the infusion duration to ensure estimation accuracy in different patient settings.

## Introduction

In recent years, the prevalence rate of multidrug-resistant gram-negative bacteria has continued to rise, and infections caused by these pathogens have emerged as a major threat to global public health (Murray et al., 2022; World Health Organization, 2022). As a last-line antibiotic for the treatment of such infections, polymyxin B has irreplaceable clinical significance (Nang et al., 2021). However, the safety profile of polymyxin B has raised concerns. Clinical data indicate that intravenous administration of polymyxin B is associated with an incidence of all-cause nephrotoxicity of 40.7% and a renal failure rate of 11.2% (Falagas et al., 2021). Moreover, the PK of polymyxin B is highly variable, and its efficacy and nephrotoxicity are exposure related (Wang et al., 2024; Zamri et al., 2025). Thus, many guidelines recommend therapeutic drug monitoring (TDM) of polymyxin B to reduce the risk of nephrotoxicity and maintain its efficacy (Tsuji et al., 2019; Abdul-Aziz et al., 2020; Liu et al., 2023; Tamma et al., 2023). Polymyxin B efficacy is best predicted by the free area under the concentration-time curve to minimum inhibitory concentration ratio (*f*AUC/MIC), which serves as the primary pharmacokinetic/pharmacodynamic (PK/PD) index (Landersdorfer et al., 2018; van der Meijden et al., 2023). And current international consensus and several clinical PK/PD studies recommend a steady-state 24-h AUC (AUC_ss,24h_) target of 50–100 mg h/L for isolates with MICs ≤ 2 mg/L, corresponding to a mean steady-state plasma concentration (C_ss,avg_) of 2–4 mg/L, to balance bactericidal activity against the risk of nephrotoxicity (Tsuji et al., 2019; Yang et al., 2022; Liu et al., 2023; Tang et al., 2023; Yu et al., 2025).

However, the determination of the polymyxin B AUC in clinical practice faces substantial practical challenges. The traditional trapezoidal method, recognized as the gold standard, offers a highly accurate depiction of the overall drug exposure profile (Neely et al., 2014). Nevertheless, it requires multiple blood samples, which complicates the procedure, compromises patient compliance, and imposes a considerable economic burden. Model-based Bayesian estimation can use only one or two samples, but selecting a suitable PPK model for specific patients is difficult. There are also difficulties in obtaining software and professional skills to perform the estimation (Liu J. et al., 2024). In contrast, the two-point method—which requires only the measurement of peak and trough concentrations—has been increasingly adopted as a practical alternative for clinical TDM, owing to its procedural simplicity and cost efficiency (Tong et al., 2025). This method is also recommended in some guidelines for polymyxin B TDM (Liu et al., 2023). However, it remains unclear whether the AUC values obtained through this streamlined approach can achieve the same level of reliability as those generated by the trapezoidal rule.

Thus, this study utilizes Monte Carlo simulations to assess the accuracy of the two-point method in estimating the polymyxin B AUC across a range of clinical scenarios. The objective of this study is to evaluate whether this simplified approach can serve as a viable alternative to the conventional trapezoidal method in clinical TDM. This study provides a theoretical foundation for the implementation of polymyxin B TDM in clinical practice.

## Methods

### PPK model selection

The literature search was performed to obtain available PPK models via PubMed and Web of Science from the inception of the database until March 2025, with the search terms “polymyxin B,” “pharmacokinetics” or “population pharmacokinetics,” “model” or “nonlinear mixed effect model” or “NONMEM”. Studies that met the following criteria were included: (1) the study population was human; (2) polymyxin B was administered by intravenous infusion; and (3) population pharmacokinetic analysis was performed, and a PPK model was developed. The exclusion criteria were as follows: (1) studies focused only on pediatric populations; (2) meta-analyses, review articles, case reports, and conference abstracts or proceedings; (3) articles that solely conducted external validation of previously published PPK models; and (4) studies with insufficient population pharmacokinetic data, such as the absence of compartmental models, covariates, parameter estimates, interindividual variability, and residual variability.

### Monte Carlo simulation and AUC estimation

A Monte Carlo simulation was conducted using NONMEM software (ICON Development Solution, Inc., USA, version 7.5.0) and R (version 4.2.0) to generate concentration–time profiles for 1,000 virtual patients under each simulated scenario. Stochastic sampling was performed using replicate-based Monte Carlo simulation with a fixed random seed (seed = 12,345) to ensure reproducibility. Patient covariates—including creatinine clearance (30, 80, and 130 mL/min) and body weight (40, 60, 80, and 100 kg)—were systematically varied to represent clinically relevant physiological heterogeneity. Polymyxin B dosing regimens were selected based on the guidelines (Tsuji et al., 2019; Liu et al., 2023) and our previously published research (Yu et al., 2022): 75 mg and 1.25 mg/kg total body weight, both administered intravenously every 12 h. Infusion durations were set at 0.5, 1, and 2 h. Inter-individual variability (IIV) was incorporated based on the variance–covariance matrices reported in the original published PPK models; whereas residual unexplained variability (RUV) was not included in the simulation.

The AUCs were estimated via two distinct methodologies: (1) AUCt, which represents true exposure calculated via the trapezoidal method (linear-up and log-down), and (2) AUCpt, derived through the two-point method on the basis of peak (immediately following infusion completion) and trough (predose) concentration measurements. The latter was computed with Equations 1, 2 as follows (Liu et al., 2023):

$$Ke=\frac{\mathrm{Ln}\left(Cp\right)-\mathrm{Ln}\left(Ct\right)}{\tau -T}$$ (1)

$$A\text{UCpt}=\left[\frac{T\times \left(\text{Cp}+\text{Ct}\right)}{2}+\frac{\left(\text{Cp}-\text{Ct}\right)}{Ke}\right]\times n$$ (2)

where Ke is the elimination constant; Cp and Ct are the peak and trough concentrations, respectively; τ is the dosing interval; T is the infusion time; and n is the number of dosages within 24 h.

### Prediction performance analysis

The prediction performance of AUCpt is evaluated via the mean absolute percent error (MAPE) as the primary metric, along with the mean percent error (MPE), root mean square error (RMSE) and normalized root mean square error (nRMSE). The calculation formulations are shown in Equations 3 –7 as follows (Tong et al., 2025; Li et al., 2022):

$$PE\%=\frac{\text{AUCpt}-\text{AUCt}}{\text{AUCt}}\times 100\%$$ (3)

$$MPE\%=\frac{1}{N}\sum_{i=1}^{N}PE\%$$ (4)

$$MAPE\%=\frac{1}{N}\sum_{i=1}^{N}\left|PE\%\right|$$ (5)

$$\text{RMSE}\%=\sqrt{\frac{1}{N}\sum_{i=1}^{N}{\left(\text{PE}\%\right)}^{2}}$$ (6)

$$nRMSE\%=\frac{RMSE\%}{\frac{1}{N}\sum_{i=1}^{N}AUCt}$$ (7)

The AUC estimation obtained via the two-point method was deemed accurate and clinically acceptable when the MAPE was within 15% or the RMSE was less than or equal to 20% (Li et al., 2022).

## Results

A total of 19 polymyxin B PPK models were included in this study, including 6 one-compartment models, 12 two-compartment models, and 1 three-compartment model. The PRISMA flow diagram illustrating the study selection process is presented in Supplementary Figure S1. The model references and PK parameter estimates are summarized in Supplementary Tables S1, S2.

**FIGURE 1:**
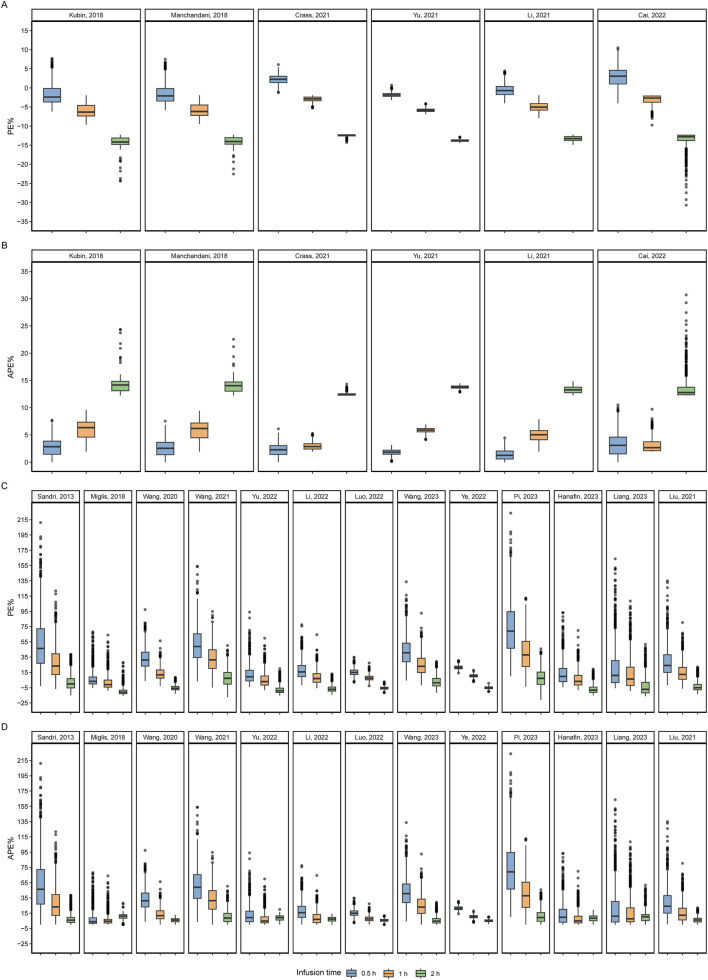
Estimation performance of various models **(A)** Predictive error of one-compartment models; **(B)** Absolute predictive error of one-compartment models; **(C)** Predictive error of multi-compartment models; **(D)** Absolute predictive error of multi -compartment models.

**FIGURE 2:**
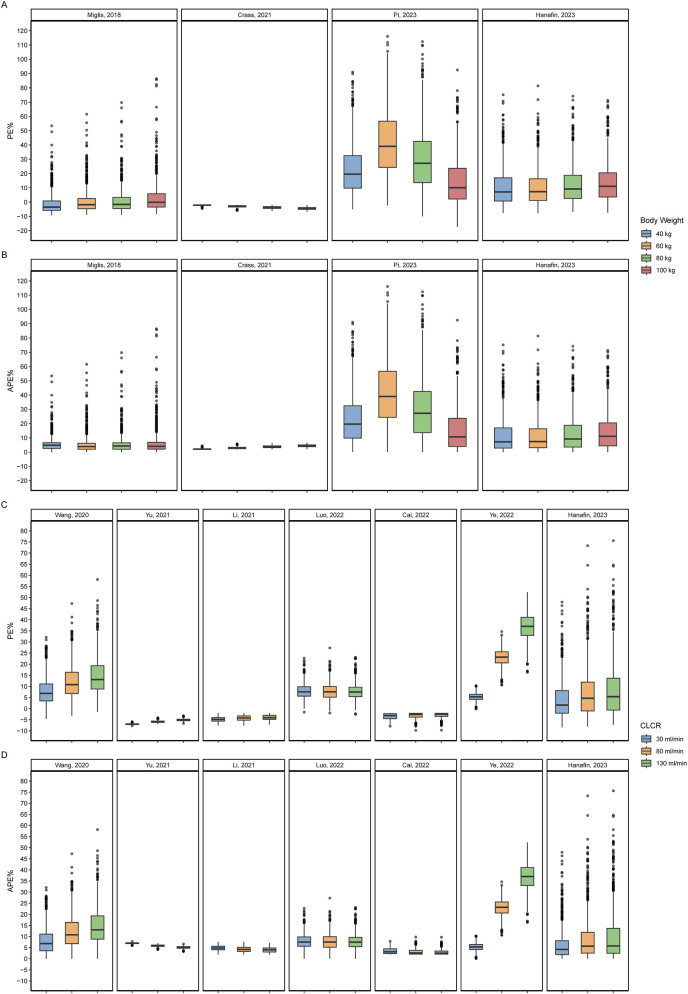
Influence of body weight and creatinine clearance on estimation accuracy **(A)** Predictive error of different body weight; **(B)** Absolute predictive error of different body weight; **(C)** Predictive error of different creatinine clearance level; **(D)** Absolute predictive error of different creatinine clearance level.

The predictive performance of the two-point method is summarized in Table 1; Figure 1. When the infusion duration was set at 0.5 h, the two-point method demonstrated favorable predictive performance across all one-compartment models. In contrast, among the two- and three-compartment models, considerable variability in the predictive performance of the two-point method was observed (MAPE ranging from 7.73% to 72.8%, and RMSE ranging from 13.0% to 80.7%), with 76.9% (10/13) of the models exhibiting low accuracy (MAPE > 15%). Notably, the predictive performance of the two-point method improved markedly with prolonged infusion time. When the infusion duration was extended to 2 h, both the MAPE and RMSE of all multicompartment models decreased to levels less than 15%. However, the opposite trend was observed in the one-compartment models, where the accuracy of the two-point method decreased significantly with increasing infusion time; nonetheless, both the MAPE and RMSE remained within acceptable limits (<15%). BW and CLCR are the main covariates of the published PPK models. A summary of the accuracy results of the two-point method under different BWs and CLCRs is presented in Figure 2. The results indicate that, for the majority of the models evaluated, BW and CLCR exert limited influences on the predictive performance of the two-point method.

**TABLE 1:** Estimation performance across varying infusion durations.

Models	MPE%			MAPE%			RMSE%			nRMSE%		
	0.5 h	1 h	2 h	0.5 h	1 h	2 h	0.5 h	1 h	2 h	0.5 h	1 h	2 h
Kubin et al. (2018)	−1.659	−5.881	−14.066	2.715	5.881	14.066	3.101	6.173	14.121	0.025	0.049	0.115
Manchandani et al. (2018)	−1.538	−5.755	−13.939	2.521	5.755	13.939	2.896	6.025	13.980	0.025	0.052	0.124
Crass et al. (2021)	2.211	−2.945	−12.465	2.239	2.945	12.465	2.508	3.025	12.467	0.017	0.021	0.086
Yu et al. (2021)	−1.802	−5.843	−13.777	1.804	5.843	13.777	1.887	5.861	13.780	0.011	0.033	0.079
Li Y. et al. (2021)	−0.613	−4.931	−13.288	1.359	4.931	13.288	1.647	5.066	13.303	0.007	0.021	0.054
Cai et al. (2022)	2.817	−3.112	−13.529	3.205	3.112	13.529	3.811	3.349	13.684	0.033	0.029	0.120
Sandri et al. (2013)	53.185	27.581	1.776	53.192	27.783	7.172	63.472	34.604	9.481	0.646	0.356	0.100
Miglis et al. (2018)	6.703	1.677	−9.511	7.733	6.013	10.328	13.001	9.127	10.959	0.152	0.108	0.129
Wang et al. (2020)	32.973	13.356	−5.295	32.973	13.380	5.657	35.608	15.545	6.249	0.217	0.096	0.039
Wang et al. (2021)	51.356	33.255	7.733	51.356	33.288	10.352	56.433	37.745	13.232	0.603	0.407	0.147
Yu et al. (2022)	12.509	5.698	−7.775	12.729	7.765	8.549	17.806	11.398	9.311	0.099	0.064	0.052
Li et al. (2022)	18.016	8.747	−6.259	18.025	9.249	6.972	21.498	12.482	7.678	0.171	0.100	0.062
Luo et al. (2022)	15.369	7.693	−5.598	15.369	7.701	5.612	16.032	8.439	5.914	0.114	0.061	0.043
Wang et al. (2022)	42.509	25.098	2.530	42.509	25.107	5.738	46.271	28.649	7.571	0.308	0.194	0.052
Ye et al. (2022)	21.476	10.418	−5.107	21.476	10.418	5.109	21.622	10.653	5.355	0.100	0.050	0.025
Pi et al. (2023)	72.802	40.786	7.931	72.802	40.793	11.239	80.659	46.768	14.421	1.273	0.759	0.246
Hanafin et al. (2023)	13.701	5.967	−6.864	13.938	8.071	8.139	20.074	11.904	9.011	0.084	0.050	0.039
Liang et al. (2023)	21.022	13.177	−3.256	21.887	15.957	10.111	34.675	24.565	12.216	0.244	0.173	0.087
Liu et al. (2021)	28.988	15.310	−3.394	28.993	15.479	6.126	34.992	19.899	7.110	0.177	0.101	0.037

Abbreviations: MPE, mean percent error; MAPE, mean absolute percent error; RMSE, root mean square error; nRMSE, normalized root mean square error.

## Discussion

To the best of our knowledge, this Monte Carlo simulation study represents the first systematic evaluation of the accuracy of the two-point method, which is based on peak and trough concentrations, to estimate the polymyxin B AUC. The findings demonstrate that the estimation performance of the two-point method is good in one-compartment models. The MAPEs in the multicompartment models were related to the infusion length, and all MAPEs were within 20% when the infusion length was prolonged to 2 h. Thus, our results support the use of the two-point method in the estimation of the polymyxin B AUC.

Currently, most published PPK studies (12/19) on polymyxin B have employed two-compartment models. The reported ranges for clearance (CL), intercompartmental clearance (Q), central volume of distribution (V1), and peripheral volume of distribution (V2) are 1.19–2.86 L/h, 1.34–13.52 L/h, 6.22–38.6 L, and 7.13–78.20 L, respectively (Wang et al., 2024). The observed discrepancies in pharmacokinetic parameters across studies may be attributed to substantial interindividual variability in polymyxin B disposition, as well as differences in the pathological-physiological status of different patient settings.

The two-point method estimates the AUC by utilizing peak and trough concentration measurements. Owing to its operational simplicity, it offers significant practical advantages in clinical TDM, particularly in resource-constrained settings. This study demonstrated that the two-point method exhibited robust predictive performance when the infusion duration was extended. These findings are consistent with results reported in previous investigations. Chen et al. demonstrated that when C_0_ and C_max_ were used to estimate the AUC of polymyxin B with a 1-h infusion duration, all deviations in AUC values fell within ±20%, with 62.5% of the cases exhibiting deviations within ±15% (Chen et al., 2021). However, this result was obtained via the one-compartment model. However, in our simulations, extending the infusion duration from 0.5 to 2 h for the one-compartment models resulted in an opposite increase in MAPE—from approximately 2%–3% to 13–14%—likely attributable to the structural absence of a distribution phase in the one-compartment models. So the peak concentration is highly sensitive to infusion duration, and a twofold change in infusion time directly alters the shape of the concentration–time profile in a region where the trapezoidal or log-linear interpolation assumptions underlying the two-point method become increasingly inaccurate. A PPK study of polymyxin B conducted in Chinese patients with multidrug-resistant gram-negative bacterial infections, which was based on a limited sampling strategy, further confirmed the high clinical applicability (r^2^ > 0.98) of the two-point model (C_0h_ + C_2h_) and its suitability for rapid therapeutic drug monitoring (Wang et al., 2020). However, this method is subject to certain limitations, particularly when applied to drugs exhibiting complex distribution dynamics.

While it demonstrates acceptable performance for one-compartment polymyxin B, the method proved unreliable in two-/three-compartment simulations under short infusions (MAPE > 15%), which may be attributed to its insufficient consideration of the drug distribution process in the multicompartment model. As is well known, Equation 2 in this manuscript is derived from the fundamental steady-state relationship of the one-compartment linear pharmacokinetic model; its application to multi-compartment drugs constitutes a simplifying approximation. In a comparative evaluation of the two-point, three-point, and four-point limited-sampling strategies for estimating the AUC_ss,24 h_ of polymyxin B following the first dose, the two-point method exhibited significantly greater mean bias relative to both the three-point and four-point methods. Under conditions of sampling time offset, its maximum bias reached −12.63% (Liu Q. et al., 2024). This systematic negative bias directly reflects the inherent defect of the two-point method based on the single-compartment model assumption, it cannot capture the rapid concentration decline in the distribution phase after infusion, resulting in an underestimation of the AUC. When polymyxin B is administered as a short (0.5 h) infusion, the drug has not yet achieved distribution equilibrium between the central and peripheral compartments at the end of infusion. As intravenous infusion duration increases, the distribution process between the central and peripheral compartments gradually approaches pseudo-equilibrium during the infusion period. With sufficiently prolonged infusion, the rapid distribution phase is substantially complete by the end of dosing, and the subsequent post-infusion concentration decline is governed predominantly by the terminal elimination phase. However, the MAPE could be improved by prolonging the infusion length. Polymyxin B has acute neurotoxicity, especially under fast infusion, according to our previous study (Liu et al., 2021). Prolonged infusion is a common option to reduce acute toxicity, and some studies have attempted 3-h infusions of polymyxin B (Yu et al., 2022). Therefore, the TDM based on peak and trough concentrations should be performed under standardized, sufficiently prolonged intravenous infusion conditions to ensure robust and reliable AUC estimation.

Optimization of the two-point method’s predictive performance for AUC is largely insensitive to BW and CLCR across most evaluated models. However, in specific models (Ye et al., 2022; Pi et al., 2023), these covariates substantially increase the estimation error of the two-point method. In the model by Pi et al. (2023), BW was included as a covariate in CL and Q using a non-standard allometric index [(BW/63.817)^2.031^ and (BW/63.817)^3.408^], resulting in a significantly greater variation in the elimination rate constant (k = CL/V) with BW compared to the model using the classical allometric scale. This led to a weight-dependent shift in the correspondence between peak and trough concentrations and AUC. While Ye et al. (2022) model included CLCR with a larger coefficient as a covariate [(CLCR/43.3)^1.22^], despite the fact that polymyxin B is mainly cleared by the non-renal pathways, this parameterization still resulted in concentration-time curves with significantly different concentrations in different renal function subgroups, increasing the estimation error of the two-point method. In contrast, other models either did not include the above covariates or used more conservative functional forms, thereby ensuring robust and consistent performance of the two-point method across all covariate strata.

On the other hand, we recognize that this study is inherently limited by the methodological constraints associated with simulation-based analyses. Although Monte Carlo simulation is a well-established and widely accepted approach in pharmacometrics for evaluating TDM strategies, it cannot fully capture the multifaceted complexity and real-world heterogeneity observed in clinical practice. Our reliance on previously published population pharmacokinetic models introduces potential methodological biases—particularly model misspecification and parameter uncertainty—which may propagate into the simulation outputs. Although we deliberately incorporated multiple PPK models derived from heterogeneous patient populations and diverse geographic settings to improve external generalizability, their predictive performance may vary substantially when applied to distinct clinical centers (Li Y.-Q. et al., 2021). Moreover, the simplified steady-state assumption, while supported by current TDM guidelines, may not adequately reflect pharmacokinetic behavior in patients with rapidly fluctuating renal function, especially during critical illness (Li Y. et al., 2021). The idealized sampling time points used in our simulations also represent a best-case scenario that may be difficult to realize with precision in clinical practice. And recent studies developing limited sampling strategies have demonstrated that even rigorously designed sampling schemes must account for inevitable timing deviations (Li et al., 2022; Pi et al., 2023).

Furthermore, the clinical efficacy of polymyxin B is best predicted by AUC/MIC rather than C_max_/MIC (Tang et al., 2023). The consensus therapeutic target of AUC_ss,24 h_ (50–100 mg h/L) can be maintained irrespective of infusion duration, as AUC is determined by dose and clearance alone. Consequently, extended-infusion regimens, which yield a lower Cmax, do not impair clinical efficacy for this AUC-dependent antibiotic. From a safety standpoint, attenuation of C_max_ may actually be advantageous. Higher peak concentrations have been associated with concentration-dependent toxicities, particularly nephrotoxicity and neurotoxicity, which represent the primary dose-limiting adverse effects of PMB (Wu et al., 2022; Liu et al., 2021; Nation et al., 2019). Therefore, prolonging the infusion duration to reduce C_max_ represents a validated strategy for mitigating polymyxin B–associated toxicity without compromising antimicrobial efficacy. Extensive antimicrobial literature further supports that prolonged infusions regimens enhance PK/PD target attainment for time-dependent agents, without requiring higher peak levels (Thabet et al., 2021).

This study had several limitations. First, owing to the Monte Carlo simulation methodology employed, the accuracy of the results is inherently dependent on the PPK model and its parameter estimates, and the findings have not been validated with real-world patient data. Translation of these simulation-based insights into clinical practice therefore necessitates prospective *in vivo* validation. Second, as a computational modeling approach, Monte Carlo simulation represents a simplification of complex clinical realities and may not fully capture the variability and unpredictability of actual clinical settings, for instance, the potential influence of suboptimal sampling timing on estimation performance was not systematically assessed. Third, the PPK models utilized in this analysis were developed and qualified in specific patient cohorts; thus, their external validity across broader or more heterogeneous clinical populations remains uncertain and warrants further evaluation. Therefore, prospective clinical studies—specifically designed to reflect real-world sampling variability—must be conducted and rigorously compared against established approaches, such as Bayesian model–guided precision dosing, to comprehensively evaluate the clinical applicability and robustness of the two-point estimation method.

## Conclusion

This study employed a Monte Carlo simulation methodology in combination with an established PPK model to evaluate the feasibility of the two-point method based on peak and trough concentration measurements. These simulation findings provide a strong pharmacokinetic rationale and generate a testable hypothesis for the clinical application of the two-point method with prolonged infusion duration. However, we suggest a prolonged infusion time of polymyxin B to reduce acute toxicity and ensure estimation accuracy in different patient settings.

## Data Availability

The original contributions presented in the study are included in the article/Supplementary Material, further inquiries can be directed to the corresponding authors.
