# Supplementary material for: Can the two-point method based on peak and trough concentrations accurately estimate the area under the curve of polymyxin B? A Monte Carlo simulation study
Source: Front Pharmacol. 2026 Jun 24;17:1838789. doi: 10.3389/fphar.2026.1838789 (PMC13341487; doi:10.3389/fphar.2026.1838789)
Supplement: Supplementary file 1 [file DataSheet1.docx]

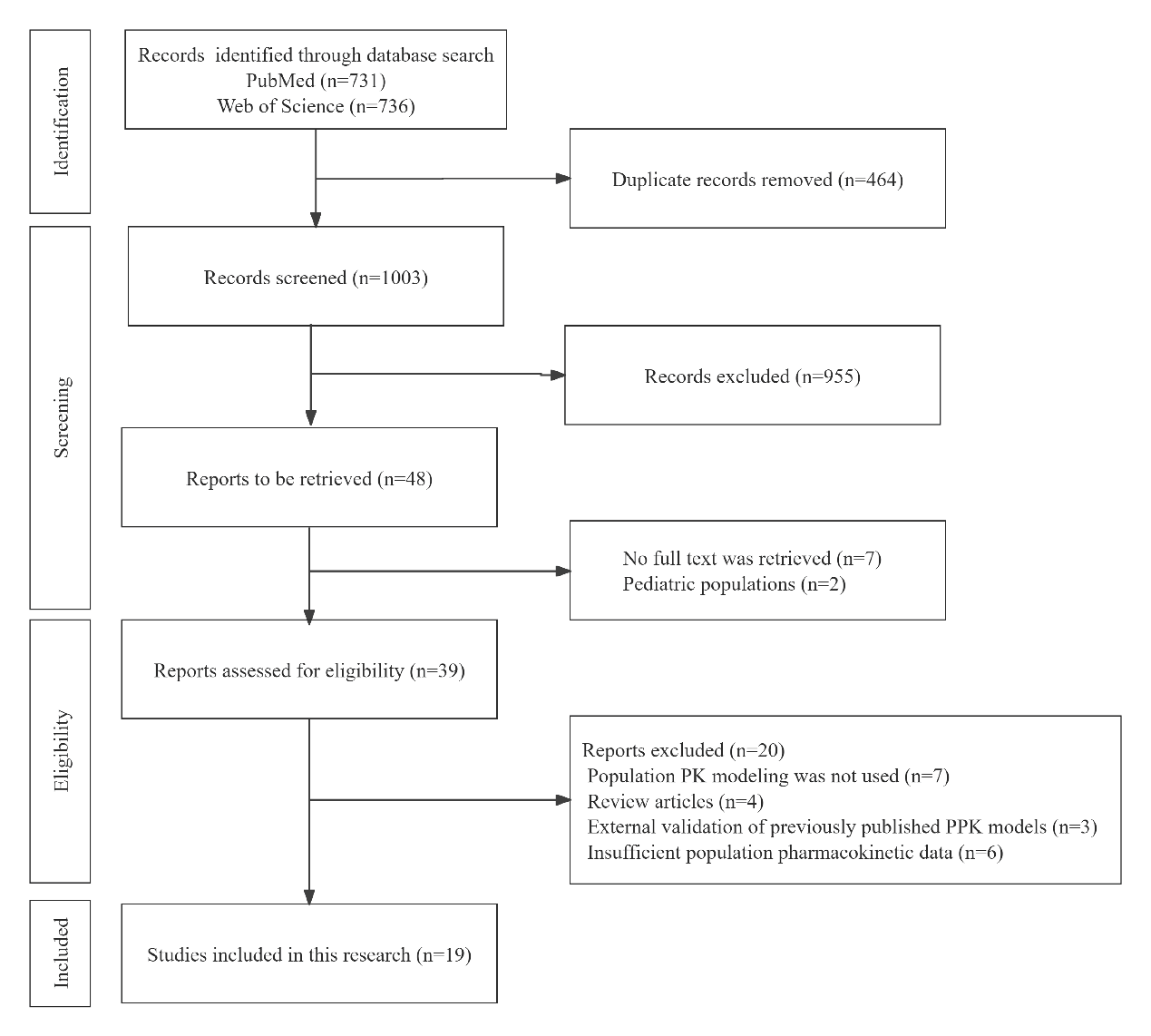


Figure 1 The PRISMA flowchart for polymyxin B PPK model selection.

Table S1 Summary of population pharmacokinetic (PK) parameters of polymyxin B

| ID | Author  (year) | Site | Study design | Subject | Software | Sampling strategy | Compartmental model | Parameter values | Interpatient variability | Residual variability |
| --- | --- | --- | --- | --- | --- | --- | --- | --- | --- | --- |
| 1 | Kubin (2018) (Kubin et al., 2018) | USA | Retrospective | multidrug-resistant Gram-negative bacterial infection patients | Monolix | Sparse | 1 | CL = 2.37  Vc = 34.4 | CL = 0.0246  Vc = 0.1421 | Additive = 0.00693  Proportional = 0.233 |
| 2 | Manchandani (2018) (Manchandani et al., 2018) | Thailand, Singapore, USA | Prospective | Adult patients with suspected / documented Gram-negative bacterial infections | ADAPT 5 | Sparse | 1 | CL = 2.5  Vc = 34.3 | CL = 0.1918  Vc = 0.2285 | NA |
| 3 | Crass (2021) (Crass et al., 2021) | USA | Prospective | Adult cystic fibrosis patients | NONMEM | Sparse | 1 | CL = 2.09  Vc = 12.7×(BW/58)^0.784 | CL = 0.0463 | Proportional = 0.188 |
| 4 | Yu (2021) (Yu et al., 2021) | China | Retrospective | Adult critically ill patients | NONMEM | Sparse | 1 | CL = 1.59×(CLCR/80)^0.408  Vc = 20.5 | CL = 0.0169 | Proportional = 0.405 |
| 5 | Li (2021) (Li et al., 2021) | China | Prospective | Adult renal transplant patients | Phoenix NLME | Sparse | 1 | CL= 1.18×(CLCR/22.2)^0.14  Vc = 12.09 | CL = 0.04  Vc = 0.06 | proportional = 0.17 |
| 6 | Cai (2022) (Cai et al., 2022) | China | Prospective | Lung transplant recipients with pneumonia | NONMEM | Sparse | 1 | CL = 1.72+(CLCR/78.49)^ 0.681  Vc = 14.4 | CL = 0.1063  Vp = 0.1648 | Proportional = 0.379 |
| 7 | Sandri (2013) (Sandri et al., 2013) | Brazil | Prospective | Critically ill patients | S-ADAPT  platform | Intensive | 2 | CL (L/h/kg) = 0.0276  Vc (L/kg) = 0.0939  Vp (L/kg) = 0.330  Q (L/h/kg) = 0.146 | CL = 0.1050  Vc = 0.5373  Vp = 0.4914  Q = 0.2540 | Additive = 0.0392  Proportional = 0.959 |
| 8 | Miglis (2018) (Miglis et al., 2018) | USA | Prospective | Adult patients without RRT, ECMO, and cystic fibrosis | PMetrics | Sparse | 2 | CL =2.63×(BW/75) ^ 0.75  Vc = 33.77  Vp = 78.20  Q = 2.32 | CL = 0.2876  Vc = 0.2028  Vp = 0.2294  Q = 0.3296 | NA |
| 9 | Wang (2020) (Wang et al., 2020) | China | Prospective | Adult patients without RRT; | Phoenix  NLME | Intensive | 2 | CL=1.79×(CLCR/105.9)^0.362  Vc = 6.22  Vp = 11.92  Q = 13.52 | CL = 0.208  Vc = 0.318  Vp = 0.690  Q = 1.508  CorrV-Cl = 0.713  CorrV-V2 = 0.667  CorrCl-V2 = 0.571 | Proportional = 0.11 |
| 10 | Wang (2021) (Wang et al., 2021) | China | Retrospective | Adult obese patients | Phoenix NLME | Intensive | 2 | CL = 2.86  Vc = 11.24  Vp = 39.70  Q = 7.36 | CL = 0.17  Vp = 1.00  Q = 0.43 | Proportional = 0.24; |
| 11 | Yu (2022) (Yu et al., 2022) | China | Prospective | Patients with bloodstream infection | NONMEM | Intensive | 2 | CL = 1.60  Vc = 38.6 × 0.371^DIS (DIS = 1 for patients and DIS = 2 for healthy subjects)  Vp = 7.13× (age/33)^1.67  Q = 3.45 | CL = 0.0331  Vc = 0.04  Vp = 0.0740  Q = 0 (fix) | Proportional = 0.118; |
| 12 | Li (2022) (Li et al., 2022) | China | Prospective | Patients without RRT; | Phoenix NLME | Sparse | 2 | CL = 2.29  Vc = 15.6  Vp = 11.8  Q = 6.81 | CL = 0.07  Vc = 0.14  Vp = 0.11  Q = 0.29 | Proportional = 0.28 |
| 13 | Luo (2022) (Luo et al., 2022) | China | Prospective | Critically ill patients with/without CRRT | NONMEM | Sparse | 2 | CL =((1-CRRT×1.5×(CLCR/83.69)^0.506 +CRRT×1.95)×exp(0.0447×(SOFA-6))  Vc = 11.7  Vp = 17.9  Q = 1.34 | CL = 0.0598  Vp = 0.854 | Proportional = 0.0589 |
| 14 | Wang (2022) (Wang et al., 2022) | China | Prospective | Elderly patients | Phoenix NLME | Sparse | 2 | CL = 1.87  Vc = 8.13 × (ALB/30.3)^1.18  Vp = 19.67  Q = 6.45 | CL = 0.12  Vc = 0.06  Vp = 0.25  Q = 0.38 | Proportional = 0.0621 |
| 15 | Ye (2022) (Ye et al., 2022) | China | Prospective | Critically ill patients with or without ECMO | NONMEM | Intensive | 2 | CL = 1.27 × (CLCR/43.3)^1.22  Vc = 8.85  Vp = 10.4  Q = 5.42 | CL = 0.0228  Vc = 0.0237  Q = 0.1102  CorrCL-Vc = 0.144 | Additive = 0.10 |
| 16 | Pi (2023) (Pi et al., 2023) | China | Prospective | Critically ill patients | Phoenix NLME | Intensive | 2 | CL=1.672×(BW/63.817)^2.031×exp[1.114 ×(CRRT = 1, not CRRT × 0)]  Vc = 7.857  Vp = 12.668  Q = 7.009 × (BW/63.817)^3.408 | CL = 0.0795  Vc = 0.1498  Vp = 0.4160  Q = 0.1267 | NA |
| 17 | Hanafin (2023) (Hanafin et al., 2023) | USA | Prospective | Adult patients, except cystic fibrosis patients, receiving concomitant inhaled PMB, or patients not anticipated to survive beyond 48 hours after starting screening | NONMEM | Intensive | 2 | CL = 1.19 × (CLCR/55.2)^0.243  Vc = 11.8 × (BW/71)  Vp = 7.92  Q = 1.98 | CL = 0.2088  Vc = 0.3931  Corr CL-Vc = 0.366 | Additive = 0.593  Proportional = 0.0767 |
| 18 | Liang (2023) (Liang et al., 2023) | China | Retrospective | Adult critically ill patients without CRRT or ECMO | Pmetrics | Intensive | 2 | CL = 1.24 × (ALB/31.45)^(−0.95)  Vc = 16.64 × (age/68)^0.95  Vp = 66.2  Q = 3.04 | CL = 0.0929  Vc = 0.5860  Vp = 0.2999  Q = 0.5592 | NA |
| 19 | Liu (2021) (Liu et al., 2021) | China | Prospective;  open-label, single-dose clinical trial | healthy Chinese subjects | NONMEM | Intensive | 3 | CL (L/h/kg) = 0.027  V1 (L/kg) = 0.071  Q2 (L/h/kg) = 0.14  V2 (L/kg) = 0.061  Q3 (L/h/kg) = 0.0064×[1+0.051×(age-28.5)]  V3 (L/kg) = 0.045×0.73^(sex) | CL = 0.0121  V1 = 0.0441  V2 = 0.0289  V3 = 0.0196 | Proportional = 0.0515 |

Abbreviations: Interindividual variability across patients was consistently quantified as variance (ω²). CL, clearance; Q, inter-compartment clearance; Vc, central volume of distribution; Vp, peripheral volume of distribution; Corr, parameter correlation; NA, not available; BW, body weight; CLCR, creatinine clearance; RRT, renal replacement therapy; ECMO, extra-corporeal membrane oxygenation; CRRT, continuous renal replacement therapy; SOFA, Sequential Organ Failure Assessment; ALB, albumin; PMB, polymin B.

**References**

Cai, X.-J., Chen, Y., Zhang, X.-S., Wang, Y.-Z., Zhou, W.-B., Zhang, C.-H., et al. (2022). Population pharmacokinetic analysis, renal safety, and dosing optimization of polymyxin B in lung transplant recipients with pneumonia: A prospective study. *Front. Pharmacol.* 13, 1019411. doi: 10.3389/fphar.2022.1019411

Crass, R. L., Al Naimi, T., Wen, B., Souza, E., Murray, S., Pai, M. P., et al. (2021). Pharmacokinetics of polymyxin B in hospitalized adults with cystic fibrosis. *Antimicrob Agents Chemother* 65, e00792-21. doi: 10.1128/AAC.00792-21

Hanafin, P. O., Kwa, A., Zavascki, A. P., Sandri, A. M., Scheetz, M. H., Kubin, C. J., et al. (2023). A population pharmacokinetic model of polymyxin B based on prospective clinical data to inform dosing in hospitalized patients. *Clinical Microbiology and Infection* 29, 1174–1181. doi: 10.1016/j.cmi.2023.05.018

Kubin, C. J., Nelson, B. C., Miglis, C., Scheetz, M. H., Rhodes, N. J., Avedissian, S. N., et al. (2018). Population pharmacokinetics of intravenous polymyxin B from clinical samples. *Antimicrob Agents Chemother* 62, e01493-17. doi: 10.1128/AAC.01493-17

Li, X., Zhang, B., Cheng, Y., Chen, M., Lin, H., Huang, B., et al. (2022). Evaluation and validation of the limited sampling strategy of polymyxin B in patients with multidrug-resistant gram-negative infection. *Pharmaceutics* 14, 2323. doi: 10.3390/pharmaceutics14112323

Li, Y., Deng, Y., Zhu, Z.-Y., Liu, Y.-P., Xu, P., Li, X., et al. (2021). Population pharmacokinetics of polymyxin B and dosage optimization in renal transplant patients. *Front. Pharmacol.* 12, 727170. doi: 10.3389/fphar.2021.727170

Liang, D., Liang, Z., Deng, G., Cen, A., Luo, D., Zhang, C., et al. (2023). Population pharmacokinetic analysis and dosing optimization of polymyxin B in critically ill patients. *Front. Pharmacol.* 14, 1122310. doi: 10.3389/fphar.2023.1122310

Liu, X., Chen, Y., Yang, H., Li, J., Yu, J., Yu, Z., et al. (2021). Acute toxicity is a dose-limiting factor for intravenous polymyxin B: A safety and pharmacokinetic study in healthy Chinese subjects. *Journal of Infection* 82, 207–215. doi: 10.1016/j.jinf.2021.01.006

Luo, X., Zhang, Y., Liang, P., Zhu, H., Li, M., Ding, X., et al. (2022). Population pharmacokinetics of polymyxin B and dosage strategy in critically ill patients with/without continuous renal replacement therapy. *European Journal of Pharmaceutical Sciences* 175, 106214. doi: 10.1016/j.ejps.2022.106214

Manchandani, P., Thamlikitkul, V., Dubrovskaya, Y., Babic, J. T., Lye, D. C., Lee, L. S., et al. (2018). Population pharmacokinetics of polymyxin B. *Clin Pharma and Therapeutics* 104, 534–538. doi: 10.1002/cpt.981

Miglis, C., Rhodes, N. J., Avedissian, S. N., Kubin, C. J., Yin, M. T., Nelson, B. C., et al. (2018). Population pharmacokinetics of polymyxin B in acutely ill adult patients. *Antimicrob Agents Chemother* 62, e01475-17. doi: 10.1128/AAC.01475-17

Pi, M.-Y., Cai, C.-J., Zuo, L.-Y., Zheng, J.-T., Zhang, M.-L., Lin, X.-B., et al. (2023). Population pharmacokinetics and limited sampling strategies of polymyxin B in critically ill patients. *J Antimicrob Chemother* 78, 792–801. doi: 10.1093/jac/dkad012

Sandri, A. M., Landersdorfer, C. B., Jacob, J., Boniatti, M. M., Dalarosa, M. G., Falci, D. R., et al. (2013). Population pharmacokinetics of intravenous polymyxin B in critically ill patients: Implications for selection of dosage regimens. *Clinical Infectious Diseases* 57, 524–531. doi: 10.1093/cid/cit334

Wang, P., Liu, D., Sun, T., Zhang, X., and Yang, J. (2022). Pharmacokinetics and pharmacodynamics of polymyxin B and proposed dosing regimens in elderly patients with multi-drug-resistant gram-negative bacterial infections. *International Journal of Antimicrobial Agents* 60, 106693. doi: 10.1016/j.ijantimicag.2022.106693

Wang, P., Zhang, Q., Feng, M., Sun, T., Yang, J., and Zhang, X. (2021). Population pharmacokinetics of polymyxin B in obese patients for resistant gram-negative infections. *Front. Pharmacol.* 12, 754844. doi: 10.3389/fphar.2021.754844

Wang, P., Zhang, Q., Zhu, Z., Feng, M., Sun, T., Yang, J., et al. (2020). Population pharmacokinetics and limited sampling strategy for therapeutic drug monitoring of polymyxin B in Chinese patients with multidrug-resistant gram-negative bacterial infections. *Front. Pharmacol.* 11, 829. doi: 10.3389/fphar.2020.00829

Ye, Q., Wang, Q., Chen, W., Zhang, R., Chen, Z., Li, P., et al. (2022). The population pharmacokinetics and dose optimization of polymyxin B in critically ill patients with or without extracorporeal membrane oxygenation. *Clinical Pharmacy Therapeu* 47, 1608–1618. doi: 10.1111/jcpt.13711

Yu, X., Jiao, Z., Zhang, C., Dai, Y., Zhou, Z., Han, L., et al. (2021). Population pharmacokinetic and optimization of polymyxin B dosing in adult patients with various renal functions. *Brit J Clinical Pharma* 87, 1869–1877. doi: 10.1111/bcp.14576

Yu, Z., Liu, X., Du, X., Chen, H., Zhao, F., Zhou, Z., et al. (2022). Pharmacokinetics/pharmacodynamics of polymyxin B in patients with bloodstream infection caused by carbapenem-resistant klebsiella pneumoniae. *Front. Pharmacol.* 13, 975066. doi: 10.3389/fphar.2022.975066

Table S2 Demographic characteristics of the included studies

| ID | Author  (year) | Site | Study design | Subject | N  (Male/Female) | PK plasma concentrations | Age | BW (kg) | CLCR (mL/min) |
| --- | --- | --- | --- | --- | --- | --- | --- | --- | --- |
| 1 | Kubin (2018) [1] | USA | Retrospective | multidrug-resistant Gram-negative bacterial infection patients | 43 (30/13) | 134 | 58 (39–69) | 78(59–95) | 73 (40–113) |
| 2 | Manchandani (2018) [2] | Thailand, Singapore, USA | Prospective | Adult patients with suspected / documented Gram-negative bacterial infections | 35 (23/12) | 139 | 58.7 ± 15.1 | 57.7 ± 15.6 | 66.8 ± 42.4 |
| 3 | Crass (2021) [3] | USA | Prospective | Adult cystic fibrosis patients | 9 (8/1) | 45 | 31 ± 12.5 | 58 ± 11.1 | 112 ± 16 |
| 4 | Yu (2021) [4] | China | Retrospective | Adult critically ill patients | 32 (26/6) | 112 | 63.63 ± 12.92 | 61.73 ± 11.77 | Scr (μmol/L), 116.69 ± 127.60 |
| 5 | Li (2021) [5] | China | Prospective | Adult renal transplant patients | 50 (32/18) | 151 | 43.5 (18–66) | 57.8 ± 12.4 | 22.2 (4.29–90.7) |
| 6 | Cai (2022) [6] | China | Prospective | Lung transplant recipients with pneumonia | 34 (25/9) | 164 | 56 ± 12.76 | 52.15 ± 10.00 | 80.81 ± 29.97 |
| 7 | Sandri (2013) [7] | Brazil | Prospective | Critically ill patients | 24 (13/11) | 192 | 61.5 (21–87) | 62.5 (41–250) | 33 (10–143) |
| 8 | Miglis (2018) [8] | USA | Prospective | Adult patients without RRT, ECMO, and cystic fibrosis | 52 (33/19) | 156 | 47 | 73 (30–122) | 68 (16–389) |
| 9 | Wang (2020) [9] | China | Prospective | Adult patients without RRT; | 46 (39/7) | 331 | 46 (18–94) | 70 (45–98) | Scr (μmol/L),  73.0 (21.0–387.0) |
| 10 | Wang (2021) [10] | China | Retrospective | Adult obese patients | 26 (17/9) | 142 | 52 (18–83) | 90 (75–125) | 84.04 (21.35–239.99) |
| 11 | Yu (2022) [11] | China | Prospective | Patients with bloodstream infection | 9 (7/2) | NA | 60 (55–65) | 68 (63–73) | 89 (68–106) |
| 12 | Li (2022) [12] | China | Prospective | Patients without RRT; | 30 (21/9) | 180 | 58.86 ± 17.01 | 58.73 ± 10.93 | 66.37 ± 45.84 |
| 13 | Luo (2022) [13] | China | Prospective | Critically ill patients with/without CRRT | Modeling group, 49 (33/16)  Validation group, 14 (9/5) | Modeling group, 147  Validation group, 42 | Modeling group, 65 (27–93)  Validation group, 61 (32–104) | Modeling group, 60 (41–91)  Validation group, 61 (48–80) | Modeling group 83.69 (13.48–197.84)  Validation group 54.06 (14.22–119.72) |
| 14 | Wang (2022) [14] | China | Prospective | Elderly patients | 23 (20/3) | 142 | 73.0 (65.0–94.0) | 70.0 (50.0–83.0) | 74.1 (17.3–200.0) |
| 15 | Ye (2022) [15] | China | Prospective | Critically ill patients with or without ECMO | 44 (33/11) | 342 | 64.1 ± 15.9 | 65.3 ± 16.5 | 43.3 (32.5–66.8) |
| 16 | Pi (2023) [16] | China | Prospective | Critically ill patients | 30 (24/6) | 212 | 55 (21–82) | 63.5 (40–90) | 104.3 ± 69.93 |
| 17 | Hanafin (2023) [17] | USA | Prospective | Adult patients, except cystic fibrosis patients, receiving concomitant inhaled PMB, or patients not anticipated to survive beyond 48 hours after starting screening | 142 (91/51) | 681 | 65 (19-94) | 71 (32.5-130) | 55.2 (8.58-322) |
| 18 | Liang (2023) [18] | China | Retrospective | Adult critically ill patients without CRRT or ECMO | 22（17/5） | 64 | 68 (31– 94) | 60(50–80) | 68.29 (34.05–192.59) |
| 19 | Liu (2021) [19] | China | Prospective;  open-label, single-dose clinical trial | healthy Chinese subjects | 20 (10/10) | NA | NA | NA | NA |

Abbreviations: BW, body weight; CLCR, creatinine clearance; Scr, serum creatinine; RRT, renal replacement therapy; ECMO, extra-corporeal membrane oxygenation; CRRT, continuous renal replacement therapy; PMB, polymin B; NA, not available.

**References**

[1] Kubin CJ, Nelson BC, Miglis C, Scheetz MH, Rhodes NJ, Avedissian SN, et al. Population Pharmacokinetics of Intravenous Polymyxin B from Clinical Samples. Antimicrob Agents Chemother 2018;62:e01493-17. https://doi.org/10.1128/AAC.01493-17.

[2] Manchandani P, Thamlikitkul V, Dubrovskaya Y, Babic JT, Lye DC, Lee LS, et al. Population Pharmacokinetics of Polymyxin B. Clin Pharma and Therapeutics 2018;104:534–8. https://doi.org/10.1002/cpt.981.

[3] Crass RL, Al Naimi T, Wen B, Souza E, Murray S, Pai MP, et al. Pharmacokinetics of Polymyxin B in Hospitalized Adults with Cystic Fibrosis. Antimicrob Agents Chemother 2021;65:e00792-21. https://doi.org/10.1128/AAC.00792-21.

[4] Yu X, Jiao Z, Zhang C, Dai Y, Zhou Z, Han L, et al. Population pharmacokinetic and optimization of polymyxin B dosing in adult patients with various renal functions. Brit J Clinical Pharma 2021;87:1869–77. https://doi.org/10.1111/bcp.14576.

[5] Li Y, Deng Y, Zhu Z-Y, Liu Y-P, Xu P, Li X, et al. Population Pharmacokinetics of Polymyxin B and Dosage Optimization in Renal Transplant Patients. Front Pharmacol 2021;12:727170. https://doi.org/10.3389/fphar.2021.727170.

[6] Cai X-J, Chen Y, Zhang X-S, Wang Y-Z, Zhou W-B, Zhang C-H, et al. Population pharmacokinetic analysis, renal safety, and dosing optimization of polymyxin B in lung transplant recipients with pneumonia: A prospective study. Front Pharmacol 2022;13:1019411. https://doi.org/10.3389/fphar.2022.1019411.

[7] Sandri AM, Landersdorfer CB, Jacob J, Boniatti MM, Dalarosa MG, Falci DR, et al. Population Pharmacokinetics of Intravenous Polymyxin B in Critically Ill Patients: Implications for Selection of Dosage Regimens. Clinical Infectious Diseases 2013;57:524–31. https://doi.org/10.1093/cid/cit334.

[8] Miglis C, Rhodes NJ, Avedissian SN, Kubin CJ, Yin MT, Nelson BC, et al. Population Pharmacokinetics of Polymyxin B in Acutely Ill Adult Patients. Antimicrob Agents Chemother 2018;62:e01475-17. https://doi.org/10.1128/AAC.01475-17.

[9] Wang P, Zhang Q, Zhu Z, Feng M, Sun T, Yang J, et al. Population Pharmacokinetics and Limited Sampling Strategy for Therapeutic Drug Monitoring of Polymyxin B in Chinese Patients With Multidrug-Resistant Gram-Negative Bacterial Infections. Front Pharmacol 2020;11:829. https://doi.org/10.3389/fphar.2020.00829.

[10] Wang P, Zhang Q, Feng M, Sun T, Yang J, Zhang X. Population Pharmacokinetics of Polymyxin B in Obese Patients for Resistant Gram-Negative Infections. Front Pharmacol 2021;12:754844. https://doi.org/10.3389/fphar.2021.754844.

[11] Yu Z, Liu X, Du X, Chen H, Zhao F, Zhou Z, et al. Pharmacokinetics/pharmacodynamics of polymyxin B in patients with bloodstream infection caused by carbapenem-resistant Klebsiella pneumoniae. Front Pharmacol 2022;13:975066. https://doi.org/10.3389/fphar.2022.975066.

[12] Li X, Zhang B, Cheng Y, Chen M, Lin H, Huang B, et al. Evaluation and Validation of the Limited Sampling Strategy of Polymyxin B in Patients with Multidrug-Resistant Gram-Negative Infection. Pharmaceutics 2022;14:2323. https://doi.org/10.3390/pharmaceutics14112323.

[13] Luo X, Zhang Y, Liang P, Zhu H, Li M, Ding X, et al. Population pharmacokinetics of polymyxin B and dosage strategy in critically ill patients with/without continuous renal replacement therapy. European Journal of Pharmaceutical Sciences 2022;175:106214. https://doi.org/10.1016/j.ejps.2022.106214.

[14] Wang P, Liu D, Sun T, Zhang X, Yang J. Pharmacokinetics and pharmacodynamics of polymyxin B and proposed dosing regimens in elderly patients with multi-drug-resistant Gram-negative bacterial infections. International Journal of Antimicrobial Agents 2022;60:106693. https://doi.org/10.1016/j.ijantimicag.2022.106693.

[15] Ye Q, Wang Q, Chen W, Zhang R, Chen Z, Li P, et al. The population pharmacokinetics and dose optimization of polymyxin B in critically ill patients with or without extracorporeal membrane oxygenation. Clinical Pharmacy Therapeu 2022;47:1608–18. https://doi.org/10.1111/jcpt.13711.

[16] Pi M-Y, Cai C-J, Zuo L-Y, Zheng J-T, Zhang M-L, Lin X-B, et al. Population pharmacokinetics and limited sampling strategies of polymyxin B in critically ill patients. Journal of Antimicrobial Chemotherapy 2023;78:792–801. https://doi.org/10.1093/jac/dkad012.

[17] Hanafin PO, Kwa A, Zavascki AP, Sandri AM, Scheetz MH, Kubin CJ, et al. A population pharmacokinetic model of polymyxin B based on prospective clinical data to inform dosing in hospitalized patients. Clinical Microbiology and Infection 2023;29:1174–81. https://doi.org/10.1016/j.cmi.2023.05.018.

[18] Liang D, Liang Z, Deng G, Cen A, Luo D, Zhang C, et al. Population pharmacokinetic analysis and dosing optimization of polymyxin B in critically ill patients. Front Pharmacol 2023;14:1122310. https://doi.org/10.3389/fphar.2023.1122310.

[19] Liu X, Chen Y, Yang H, Li J, Yu J, Yu Z, et al. Acute toxicity is a dose-limiting factor for intravenous polymyxin B: A safety and pharmacokinetic study in healthy Chinese subjects. Journal of Infection 2021;82:207–15. https://doi.org/10.1016/j.jinf.2021.01.006.
